# Supplementary material for: Observations about utilitarian coherence in the avian compass
Source: Sci Rep. 2022 Apr 9;12:6011. doi: 10.1038/s41598-022-09901-7 (PMC8994785; doi:10.1038/s41598-022-09901-7)
Supplement: Supplementary file 1 — Supplementary Information. [file 41598_2022_9901_MOESM1_ESM.pdf]

Supplementary Information for

Observations about utilitarian coherence in the avian compass

Luke D. Smith, Jean Deviers and Daniel R. Kattnig

Daniel R. Kattnig.
E-mail: d.r.kattnig@exeter.ac.uk

This PDF file includes:

Supplementary Methods
Supplementary Discussion
Figs. S1 to S8
Tables S1 to S4
Code Snippets
SI References

Supplementary Methods

Calculation of recombination yields for variable relative reorientation of radicals. The master equation

$$\begin{aligned}\frac{\mathrm{d}\hat{\rho}(t)}{\mathrm{d}t} &= -i\left[\hat{H},\hat{\rho}(t)\right] - \frac{k_S}{2}\left\{\hat{P}_S,\hat{\rho}(t)\right\} - \frac{k_T}{2}\left\{\hat{P}_T,\hat{\rho}(t)\right\} \\ &= -i\left[\hat{H},\hat{\rho}(t)\right] - k\hat{\rho}(t),\end{aligned} \quad [S1]$$

with $\hat{H} = \hat{H}_\mathrm{A} + \hat{H}_\mathrm{B}$, is opportune insofar as it allows one to express the singlet yield of the recombination reaction in terms of the spin correlation tensors (SCTs), $\mathbf{T}^{(i)}_{\alpha,\beta}(t)$, with components (1–3)

$$\begin{aligned}T^{(i)}_{\alpha,\beta}(t) &= \frac{1}{z_i}\mathrm{Tr}[\hat{S}_{i,\alpha}\hat{S}_{i,\beta}(t)] \\ &= \frac{1}{z_i}\mathrm{Tr}[\hat{S}_{i,\alpha}e^{i\hat{H}_i t}\hat{S}_{i,\beta}e^{-i\hat{H}_i t}],\end{aligned} \quad [S2]$$

where z_i denotes the dimension of the nuclear subspace of the Hilbert space of radical i. Specifically, the singlet yield of a radical pair born in the singlet configuration, $\hat{\rho}(0) = \hat{P}_S/(z_1 z_2)$, is given by

$$Y_\mathrm{S} = k\int_0^\infty p_S(t)\exp\left(-kt\right)\,\mathrm{d}t, \quad [S3]$$

where

$$\begin{aligned}p_S(t) &= \frac{1}{z_A z_B}\mathrm{Tr}[\hat{P}_S\hat{P}_S(t;k=0)] \\ &= \frac{1}{4} + \sum_{\alpha,\beta} T^{(A)}_{\alpha,\beta}(t)T^{(B)}_{\alpha,\beta}(t).\end{aligned} \quad [S4]$$

Here, $\hat{P}_S(t;k=0)$ is the singlet projection operator in the Heisenberg picture evaluated for $k = 0$. As $\mathbf{T}^{(i)}(t)$ is defined for the individual radicals, i.e., calculable within the individual Hilbert space of radical i, equations (S2) to (S4) allow the treatment of radical pairs with a reasonably complex hyperfine coupling pattern involving many coupled nuclei without common symmetry of the interactions, as expected for the radicals implicated with the magnetoreception processes. Furthermore, if the SCTs are available on a grid of magnetic field orientations (spanning a hemisphere), the SCTs of radicals that have been rotated (by rotation matrix $\mathbf{R}$) can be efficiently reconstructed from the unrotated SCTs due to the property (1).

$$\mathbf{T}^{(i)}(t;\vec{B}_0,\{\mathbf{R}\mathbf{A}_{i,j}\mathbf{R}^{-1}\}) = \mathbf{R}\mathbf{T}^{(i)}(t;\mathbf{R}^{-1}\vec{B}_0,\{\mathbf{A}_{i,j}\})\mathbf{R}^{-1} \quad [S5]$$

Here, $\mathbf{T}^{(i)}(t;\vec{B}_0,\{\mathbf{A}_{i,j}\})$ denotes the spin correlation tensor for a given magnetic field, $\vec{B}_0$, and set of hyperfine tensor parameters, $\{\mathbf{A}_{i,j}\}$, and $\mathbf{T}^{(i)}(t;\mathbf{R}^{-1}\vec{B}_0,\{\mathbf{A}_{i,j}\})$ the spin correlation tensor of the radical with rotated orientation. Equation (S5) allows the efficient evaluation of the singlet yield of complex radical pairs as a function of their mutual orientation in space, as has been demonstrated in a previous study (1). Here, we will adapt this approach to evaluate the dependence of coherence measures and compass fidelity on relative radical pair orientation.

To this end, observe that for $k = 0$ the electronic spin density operator $\hat{\sigma}(t) = \mathrm{Tr}_n\left[\hat{\rho}(t)\right]$ (with Tr_n denoting the trace over all nuclear spins of both radicals) can be expressed in terms of $\mathbf{T}^{(i)}(t)$, i.e.

$$\hat{\sigma}(t) = \frac{\hat{1}}{4} + \sum_{\alpha,\beta\in\{x,y,z\}} c_{\alpha\beta}(t)\hat{S}_{A,\alpha}\hat{S}_{B,\beta}, \quad [S6]$$

where

$$c_{\alpha\beta}(t) = -4\sum_{\gamma\in\{x,y,z\}} T^{(A)}_{\gamma\alpha}(t)T^{(B)}_{\gamma\beta}(t). \quad [S7]$$

Equations (S5) and (S6) thus provide a means to calculate electronic coherence quantifiers and singlet recombination yields of complex radical systems subject to arbitrary relative reorientations of the constituent radicals.

Hyperfine coupling parameters and simulation details. Hyperfine interaction parameters are chosen in accordance with DFT calculations employing the UB3LYP/6-31+G(d,p)//UB3LYP/EPR-III protocol, for which more details may be found in the SI of a previous study (1). The hyperfine coupling parameters are shown for: the flavin anion radical in Supplementary Table S1, the tryptophan radical cation in Supplementary Table S2, and the neutral tyrosine radical in Supplementary Table S3. Together these provide the hyperfine couplings for a total of 21 nuclei in flavin-tryptophan, and 19 nuclei in flavin-tyrosine radical pairs. With these parameters fixed we sampled 878,400 relative orientations of the radicals as described in the main text. Spin correlation tensors were calculated using GPU parallel processing with CUDA (NVidia Tesla K80 GPU and Intel Xeon CPU E5-2640v3 @ 2.60GHz), which provided a 6.5-time speedup over our central processing unit (CPU) implementation for calculating the spin correlation tensors of the flavin anion radical using double precision arithmetics throughout. Further details are described in Ref. (1). Hyperfine parameters of N5 and N10 associated with flavin, used in the 5 nuclear spin system studies conducted, are also shown in Supplementary Table S1.

Randomly selected hyperfine coupling. For the studies presented on systems with 5 nuclear spins, some hyperfine coupling parameters are randomly sampled. The random sampling is conducted for a minimum of 2400 instances per system such that the isotropic hyperfine coupling constant $|a_{iso}| \leq 28$ MHz and hyperfine tensor in its principal orientation

$$A_{ij} = \left[a_{iso} - \frac{\delta}{3\,(1+\eta)}, a_{iso} - \frac{\delta}{3\,(1-\eta)}, a_{iso} + \frac{2\delta}{3}\right], \tag{S8}$$

where $0 \leq \eta \leq 1$, $0 \leq \delta \leq 28$ MHz for the case of $k^{-1} = 1$ μs and $0 \leq \delta \leq 14$ MHz for $k^{-1} = 10$ μs. All random variables were sampled from a uniform distribution over their respective domain. Subsequently, the tensors were rotated via random unitary matrices such that the hyperfine coupling is given by $U^T A_{ij} U$.

Calculation of large spin system global coherence measure. In order to evaluate the coherence measure

$$[\mathcal{C}_y^{\mathcal{G}}]_{B=0} = \left| Y_S \left(\hat{\rho}(0) = \frac{\mathbb{GC}\left(\hat{P}_S\right)}{z_1 z_2} \right) \right|, \tag{S9}$$

as introduced by Cai and Plenio (4), we proceeded as follows. In the eigenbasis of the Hamiltonian, the recombination yield of a singlet-born radical pair, $\hat{\rho}\,(0) = \hat{P}_S/Z$, is given by (5)

$$\begin{aligned} Y_S \left(\hat{\rho}\,(0) = \frac{\hat{P}_S}{Z} \right) &= \frac{k^2}{Z} \sum\nolimits_{i,j} \frac{\left| \langle i \left| \hat{P}_S \right| j \rangle \right|^2}{\Delta\omega_{i,j}^2 + k^2} \\ &= \frac{1}{4} + \frac{k^2}{Z} \sum\nolimits_{i,j} \frac{\left| \langle i \left| \hat{\mathbf{S}}_1 \cdot \hat{\mathbf{S}}_2 \right| j \rangle \right|^2}{\Delta\omega_{i,j}^2 + k^2}, \end{aligned} \tag{S10}$$

where $Z = z_1 z_2$ and $\Delta\omega_{i,j} = \omega_i - \omega_j$ with ω_i denoting the eigenvalue of $\hat{H}$ associated with eigenstate $|i\rangle$. On the other hand, for the incoherent initial state, $\hat{\rho}(0) = Z^{-1}\mathbb{IC}(\hat{P}_S) = Z^{-1} \sum_i |i\rangle\langle i|\hat{P}_S|i\rangle\langle i|$, the singlet recombination yield is easily evaluated as

$$\begin{aligned} Y_S\left(\hat{\rho}\,(0) = Z^{-1}\mathbb{IC}\left(\hat{P}_S\right)\right) &= \frac{1}{Z} \sum\nolimits_i \left| \langle i \left| \hat{P}_S \right| i \rangle \right|^2 \\ &= \frac{1}{4} + \frac{1}{Z} \sum_i \left| \langle i \left| \hat{\mathbf{S}}_1 \cdot \hat{\mathbf{S}}_2 \right| i \rangle \right|^2. \end{aligned} \tag{S11}$$

The second equality for both eq. (S10) and (S11) results from the property of the Cartesian spin operators that $\mathrm{Tr}\left[\hat{S}_{i,\alpha}\right] = 0$ ($\alpha \in \{x, y, z\}$) together with the explicit expression $\hat{P}_S = \frac{\hat{1}}{4} - \hat{\mathbf{S}}_1 \cdot \hat{\mathbf{S}}_2$. Because of the linearity of the equation of motion, the coherence measure as introduced in eq. (S9) is simply obtained as

$$[\mathcal{C}_y^{\mathcal{G}}]_{B=0} = \frac{k^2}{Z} \sum\nolimits_{i \neq j} \frac{\left| \langle i \left| \hat{\mathbf{S}}_1 \cdot \hat{\mathbf{S}}_2 \right| j \rangle \right|^2}{\Delta\omega_{i,j}^2 + k^2}. \tag{S12}$$

For spin systems studied here, $\hat{\mathbf{S}}_1 \cdot \hat{\mathbf{S}}_2$ or $\hat{P}_S$ are prohibitively large to be explicitly assembled in computer memory and evaluation of the yield is computationally demanding as it scales quadratically with the (huge) composite Hilbert space dimension. To circumvent the former issue, we express $[\mathcal{C}_y^{\mathcal{G}}]_{B=0}$ in terms of the Cartesian spin operators $\hat{S}_{i,\alpha}$ as follows

$$[\mathcal{C}_y^{\mathcal{G}}]_{B=0} = \frac{k^2}{Z} \sum\nolimits_{\text{offdiag}} \frac{\left| \sum_{\alpha \in \{x,y,z\}} \langle i_1 \left| \hat{S}_{1,\alpha} \right| j_1 \rangle \langle i_2 \left| \hat{S}_{2,\alpha} \right| j_2 \rangle \right|^2}{\left(\Delta\omega_{i_1,j_1} + \Delta\omega_{i_2,j_2}\right)^2 + k^2}, \tag{S13}$$

where $|i_1\rangle$ and $|i_2\rangle$ are the eigenstates of the (individual) Hamiltonians of radicals A and B, respectively, and the sum extends over all associated states except those simultaneously obeying $i_1 = j_1$ and $i_2 = j_2$. This approach, which is possible here as inter-radical interactions have been neglected, i.e., $\hat{H} = \hat{H}_1 + \hat{H}_2$, requires only the representation matrices of the Cartesian components of the electron spin operators in their respective radical Hilbert space. In order to evaluate $[\mathcal{C}_y^{\mathcal{G}}]_{B=0}$ in a time-efficient manner we further substitute the sum in eq. (S13) by a Monte-Carlo estimate obtained as $d\,(d-1)$, with d denoting the combined Hilbert space dimension, times the average value of the summand evaluated for N random samples of the index quadruples i_1, i_2, j_1 and j_2, subject to the above-stated constraint of excluding diagonal contributions:

$$[\mathcal{C}_y^{\mathcal{G}}]_{B=0} \approx k^2 \frac{4\,(d-1)}{N} \sum\nolimits_{\text{random offdiag}} \frac{\left| \sum_{\alpha \in \{x,y,z\}} \langle i_1 \left| \hat{S}_{1,\alpha} \right| j_1 \rangle \langle i_2 \left| \hat{S}_{2,\alpha} \rangle \right| j_2 \right|^2}{\left(\Delta\omega_{i_1,j_1} + \Delta\omega_{i_2,j_2}\right)^2 + k^2}. \tag{S14}$$

For the data shown in Fig. 5 in the main document we have used 10^9 index samples for the evaluation of $[\mathcal{C}_y^{\mathcal{G}}]_{B=0}$ for every considered relative orientation of the radical pair. To this end, eq. (S14) was implemented in Fortran making use of multi-threading (via OpenMP) to efficiently sample the sum and interfaced to Python using F2PY. The eigenvalues/eigenvectors

were computed using LAPACK routine zheevd as implemented in the Intel Math Kernel Library (version 2020.0.2). Results were compared to the complete sampling implemented on CPU and GPU, with the latter offering a 40-times speed up. The MC sampling as implemented above gives rise to deviations of $[\mathcal{C}_y^{\mathcal{G}}]_{B=0}$ from the true value of the order of 0.0001, which is sufficiently small relative to the spread of coherence measures relevant for the systems studied. GPU and CPU result differed by 10-9 due to the noncommutativity of floating point additions and different numbers of accumulators/threads used in the CPU and GPU implementations.

Additionally, it is interesting to note that the radicals studied here comprised an odd number of coupled hydrogen atoms and a non-degenerate spectrum. As a consequence of time-reversal symmetry of the Hamiltonian for $B = 0$, the diagonal contribution to the yield, eq. (S10), evaluates to 1/4 exactly. See e.g. the supporting information of (6) for details.

Supplementary Discussion

5 nuclear spin system electronic and global coherence measures. As in the main text, we have considered correlations of coherence with anisotropy for several mean electronic coherence measures over extremal orientations and compared to the global coherence measure $[\mathcal{C}_y^{\mathcal{G}}]_{B=0}$. To assess our measures in the small system regime, we elected to focus on systems with 5 randomly chosen hyperfine interactions, whereby we partly retained the dominant hyperfine interactions of the flavin N5 and N10 nuclei as these are considered essential to the processes. Table S4 summarizes the various systems studied, which will be referred to via labels A to H. In Supplementary Fig. S5 correlations for a wide range of measures are shown, such as $\Delta[\bar{\mathcal{C}}_i]$, and a field independent measure $[\bar{\mathcal{C}}_i]_{B=0}$. These measures are also presented for global coherences using $\mathcal{C}_y^{\mathcal{G}}$ and $\bar{\mathcal{C}}_{l_1}^{\mathcal{G}}$. Similar results for correlations are shown in Supplementary Fig. S6 using the relative anisotropy (Γ_S), defined as

$$\Gamma_S = \frac{\Delta_S}{\overline{Y}_S}, \tag{S15}$$

where the mean quantum yield is given by

$$\overline{Y}_S = \frac{1}{4\pi}\int_0^{\pi} \mathrm{d}\vartheta \int_0^{2\pi} \mathrm{d}\varphi \, \sin(\vartheta) Y_S(\vartheta, \varphi). \tag{S16}$$

Several features are identified through analysing a wide range of measures. For example, with respect to global measures, we find that an increase in global coherence does not necessarily signify an increase in anisotropy. This is demonstrated by the measures $\mu[\bar{\mathcal{C}}_{l_1}^{\mathcal{G}}]$ and $[\bar{\mathcal{C}}_{l_1}^{\mathcal{G}}]_{B=0}$, which show an anticorrelation with anisotropy. In contrast, the results show that the global coherence quantifier $\mathcal{C}_y^{\mathcal{G}}$ correlates with anisotropy, but the degree of correlation is system dependent. The measure is more effective when evaluated for $B = 0$, as originally perceived, than for the actual applied field. This further supports our finding that it is important that the global coherence quantifier relates to operation.

Furthermore, for systems with hyperfine couplings only within one of the radicals (E - H), a larger correlation is found with electronic coherence measures. With respect to $\Delta[\bar{\mathcal{C}}_i]$, the correlation is more system dependent. For systems, E and F, which contain N5 and N10 in addition to random hyperfine interactions, $\Delta[\bar{\mathcal{C}}_i]$ exhibits strong anticorrelation suggesting an advantage to a greater amount of coherence for the orientation associated with minimum singlet yield for these systems. On the other hand, for systems G and H, which use entirely random hyperfine couplings, $\Delta[\bar{\mathcal{C}}_i]$ is shown to have a low correlation for most measures except for $\Delta[\bar{\mathcal{C}}_{st}^{\mathcal{E}}]$. The opposite can be said for measures $[\bar{\mathcal{C}}_i]_{B=0}$ for which $[\bar{\mathcal{C}}_{st}^{\mathcal{E}}]_{B=0}$ presents a low correlation. This suggests that the measure $\Delta[\bar{\mathcal{C}}_{st}^{\mathcal{E}}]$ may have an advantage in identifying coherence-anisotropy correlations for some systems of reference-probe topology.

To illustrate these points further the scatter and correlation data of relevant examples of system G is shown in Supplementary Fig. S7, where system C has been included for comparison. The results for system G show that accounting for global coherence via $[\mathcal{C}_{l_1}^{\mathcal{G}}]_{B=0}$ does not identify coherence as a resource for sensitivity. In contrast, $[\mathcal{C}_y^{\mathcal{G}}]_{B=0}$, which is directly based on the singlet yield, presents global coherence as a resource. The electronic coherence correlation data shows that whilst both $\mathcal{C}_r^{\mathcal{E},ST}$ and $\mathcal{C}_{st}^{\mathcal{E}}$ find strong correlation with anisotropy depending on the derived measure used, they report on different features. As $[\bar{\mathcal{C}}_{st}^{\mathcal{E}}]_{B=0}$ is near zero and has almost no correlation to anisotropy, the stronger correlations observed for $\Delta[\bar{\mathcal{C}}_{st}^{\mathcal{E}}]$ and $\mu[\bar{\mathcal{C}}_{st}^{\mathcal{E}}]$ are realized to be generated as a direct result of the magnetic field interaction. This highlights the need for both an operational interpretation considering both nuclear and electronic parts and the value of (electronic) measures to highlight specific behaviour, although the latter appears to not be universal but system dependent. In Supplementary Fig. S8 relative entropy electronic coherence measures are compared against the global coherence measure for system A and system B, which is equivalent to system A, but with a longer radical lifetime of $k^{-1} = 10\,\mu$s. For these systems it is demonstrated that a longer lifetime is associated with a decrease in global coherence and its correlation with anisotropy. Conversely, there is a small rise in the correlation with electronic coherence for the longer lifetime. This behaviour is not observed in the reference-probe type systems E-H.

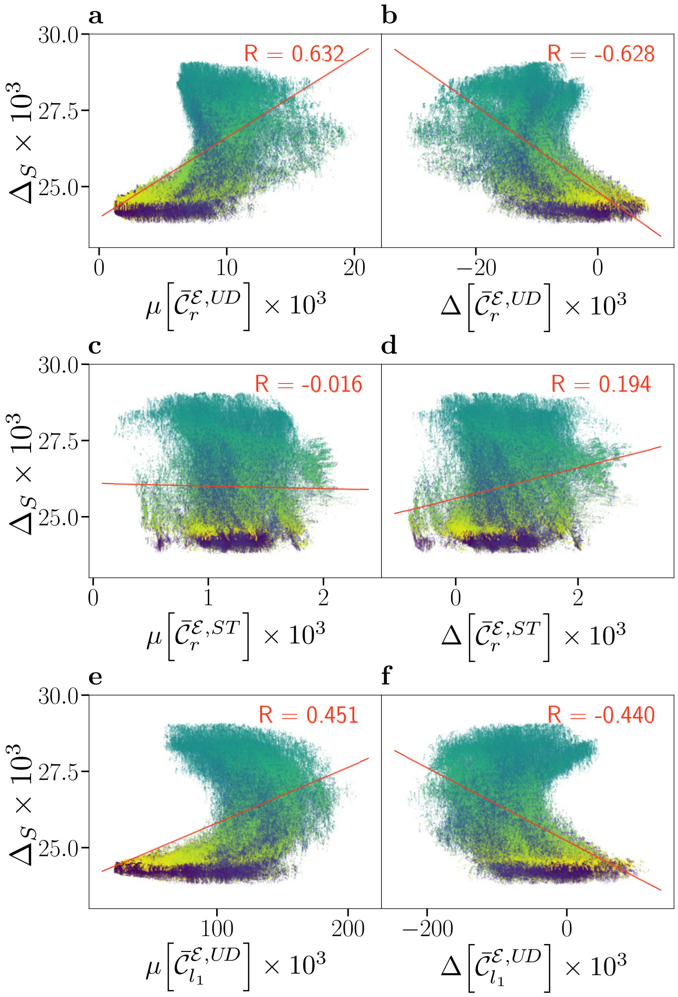

Fig. S1. Compass sensitivity, as captured by the anisotropy Δ_S, is plotted against electronic coherence measures $\mu[\bar{\mathcal{C}}_i]$ (**a**, **c**, **e**) and $\Delta[\bar{\mathcal{C}}_i]$ (**b**, **d**, **f**), for $878, 400$ relative orientations of flavin-tryptophan radical pairs. The magnetic field strength is chosen as 1 mT. Data has been coloured according to the relative orientation angle β, ranging from 0° (blue) to 180° (yellow). Both up-down (UD) and singlet-triplet (ST) basis are considered, and a linear fit (red line) with associated Pearson correlation coefficient R is displayed.

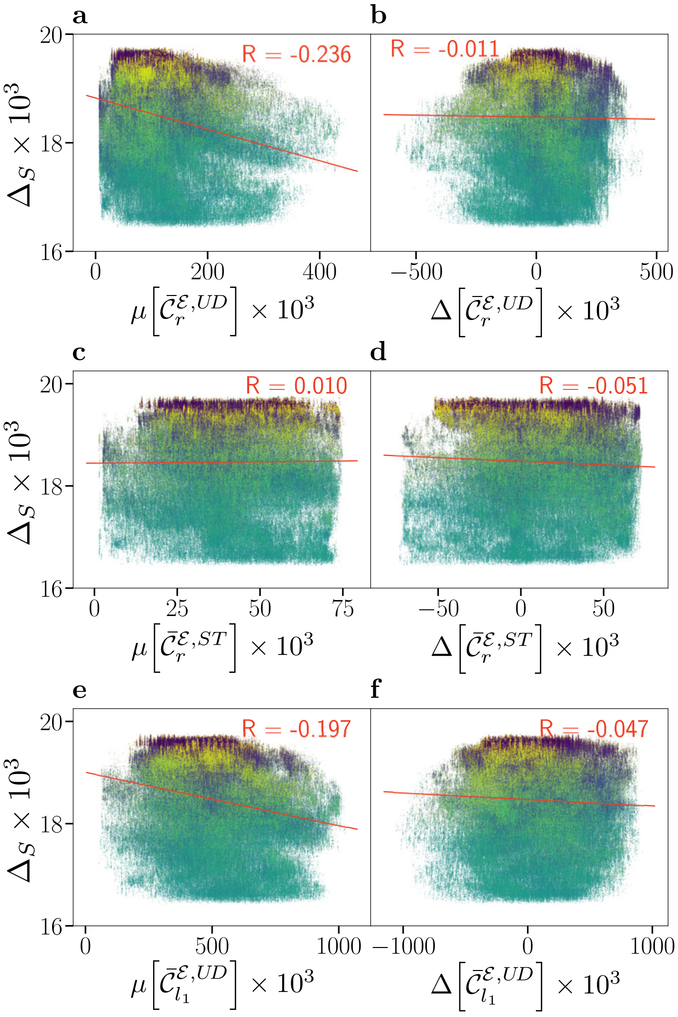

Fig. S2. Compass sensitivity, as captured by the anisotropy Δ_S, is plotted against electronic coherence measures $\mu[\bar{\mathcal{C}}_i]$ (**a**, **c**, **e**) and $\Delta[\bar{\mathcal{C}}_i]$ (**b**, **d**, **f**), for $878,400$ relative orientations of flavin-tryptophan radical pairs. The magnetic field strength is chosen as 5 mT. Data has been coloured according to the relative orientation angle β, ranging from 0° (blue) to 180° (yellow). Both up-down (UD) and singlet-triplet (ST) basis are considered, and a linear fit (red line) with associated Pearson correlation coefficient R is displayed.

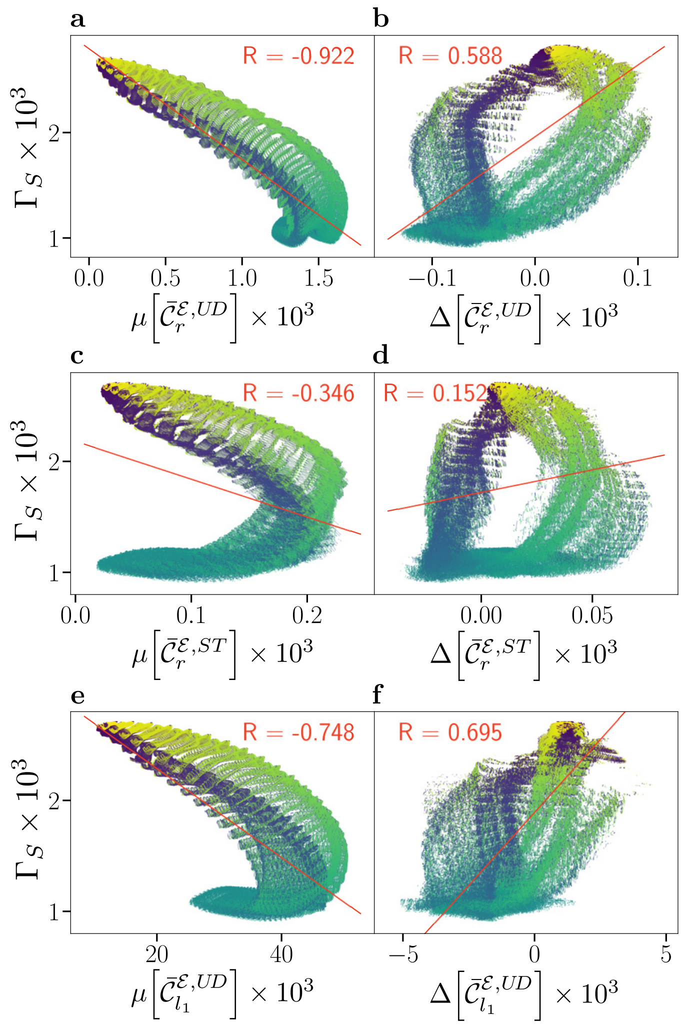

Fig. S3. Compass sensitivity, as captured by the relative anisotropy Γ_S, is plotted against electronic coherence measures $\mu[\bar{\mathcal{C}}_i]$ (**a**, **c**, **e**) and $\Delta[\bar{\mathcal{C}}_i]$ (**b**, **d**, **f**), for $878, 400$ relative orientations of flavin-tryptophan radical pairs assuming B = $50\,\mu$T. Data has been coloured according to the relative orientation angle β, ranging from 0° (blue) to 180° (yellow). Both up-down (UD) and singlet-triplet (ST) basis are considered, and a linear fit (red line) with associated Pearson correlation coefficient R is displayed.

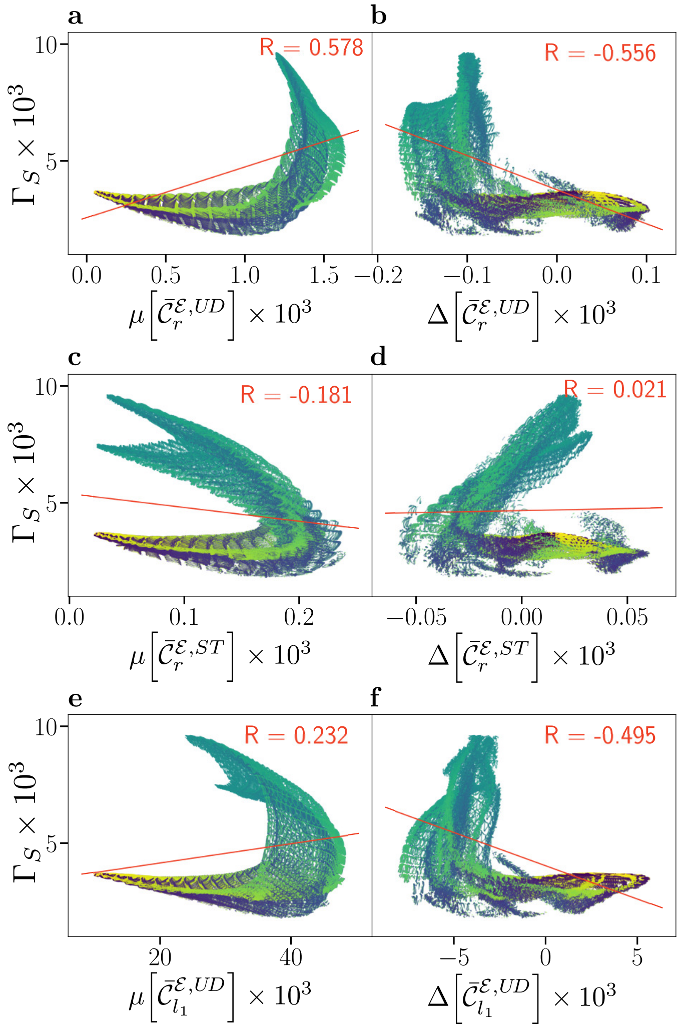

Fig. S4. Compass sensitivity, as captured by the relative anisotropy Γ_S, is plotted against electronic coherence measures $\mu[\bar{\mathcal{C}}_i]$ (**a**, **c**, **e**) and $\Delta[\bar{\mathcal{C}}_i]$ (**b**, **d**, **f**), for $878,400$ relative orientations of flavin-tyrosine radical pairs assuming B = $50\,\mu$T. Data has been coloured according to the relative orientation angle β, ranging from 0° (blue) to 180° (yellow). Both up-down (UD) and singlet-triplet (ST) basis are considered, and a linear fit (red line) with associated Pearson correlation coefficient R is displayed.

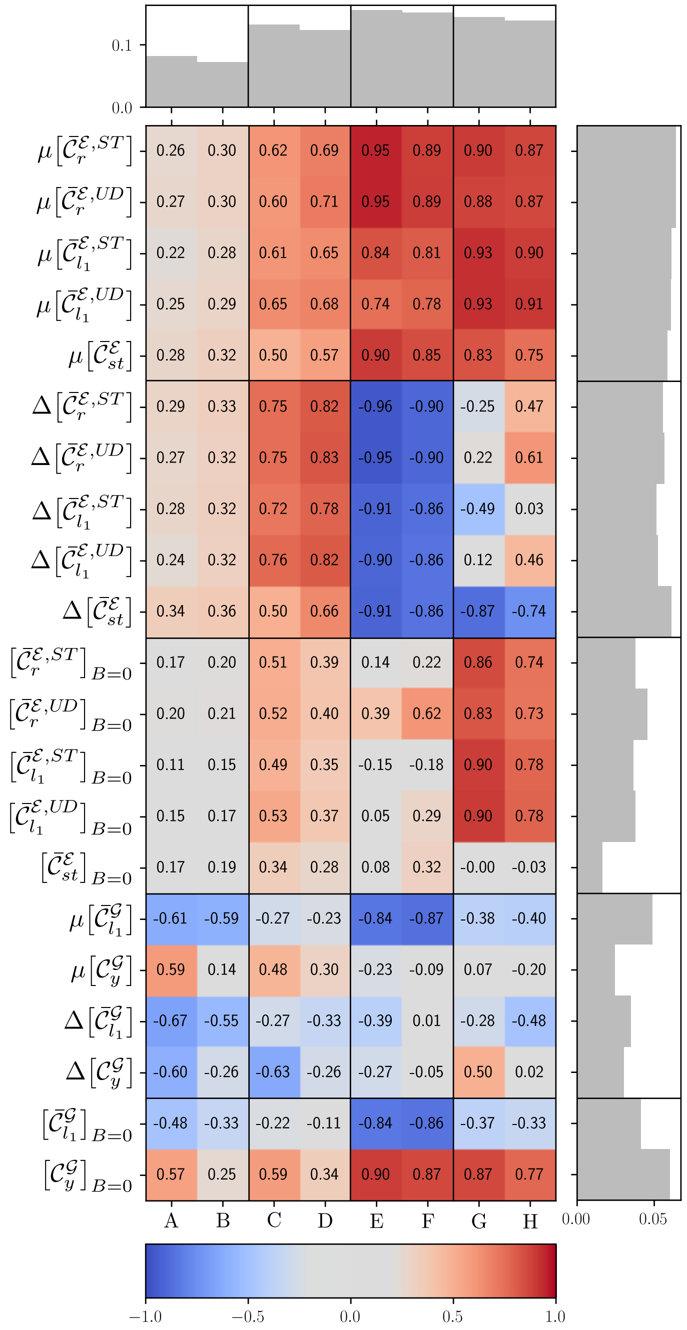

Fig. S5. Correlation coefficients between anisotropy Δ_S and coherence measures are plotted for a range of measures, shown on the y-axis, and for 8 system configurations (shown in Table. S4 for systems A-H), each comprising 5 nuclear spins. Bar plots at the top and at the right side represent the fraction of the sum of absolute values of the correlation coefficients within the plot, for a respective column or row. Black lines partitioning columns group together similar systems. The first column entry of a vertical partition represents the system for $k^{-1} = 1\,\mu$s and the second column entry represents the same system with the longer lifetime of $k^{-1} = 10\,\mu$s. Likewise, the row partition separates the different electronic measures and the global measure.

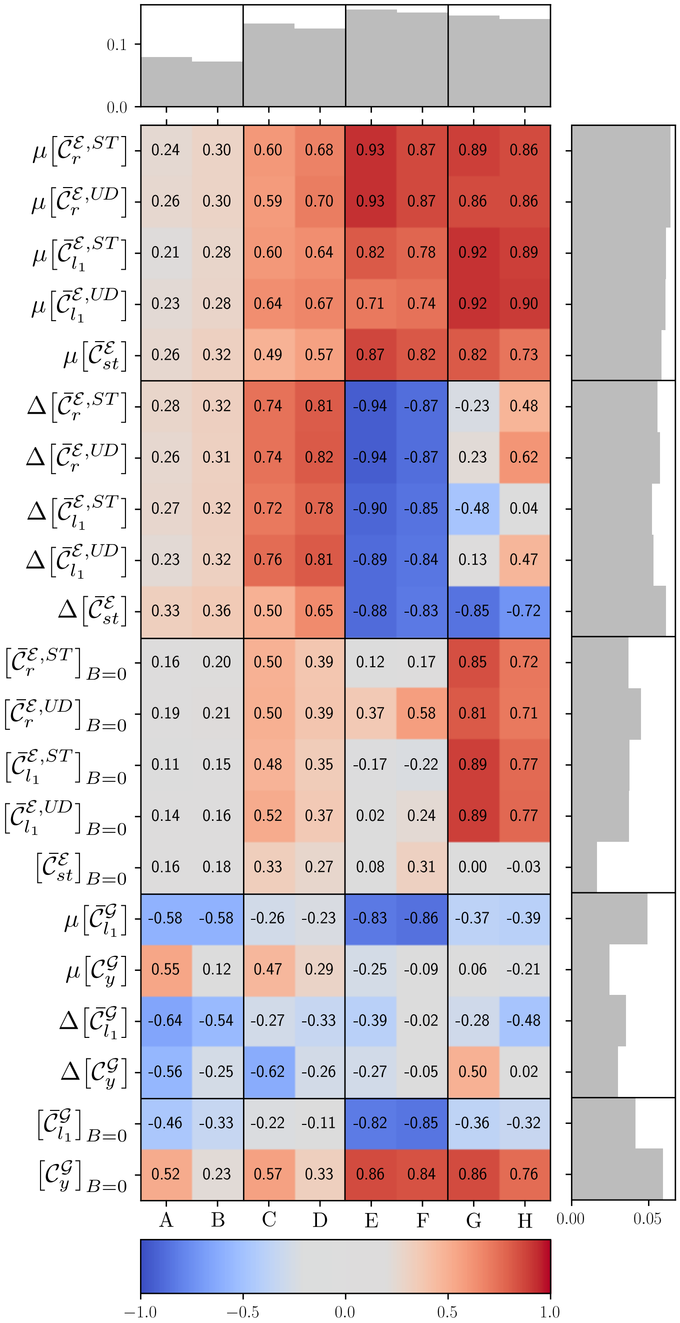

Fig. S6. Correlation coefficients between relative anisotropy Γ_S and coherence measures are plotted for a range of measures, shown on the y-axis, and for 8 system configurations (shown in Table. S4 for systems A-H), each comprising 5 nuclear spins. Bar plots at the top and at the right side represent the fraction of the sum of absolute values of the correlation coefficients within the plot, for a respective column or row. Black lines partitioning columns group together similar systems. The first column entry of a vertical partition represents the system for $k^{-1} = 1\,\mu$s and the second column entry represents the same system with the longer lifetime of $k^{-1} = 10\,\mu$s. Likewise, the row partition separates the different electronic measures and the global measure.

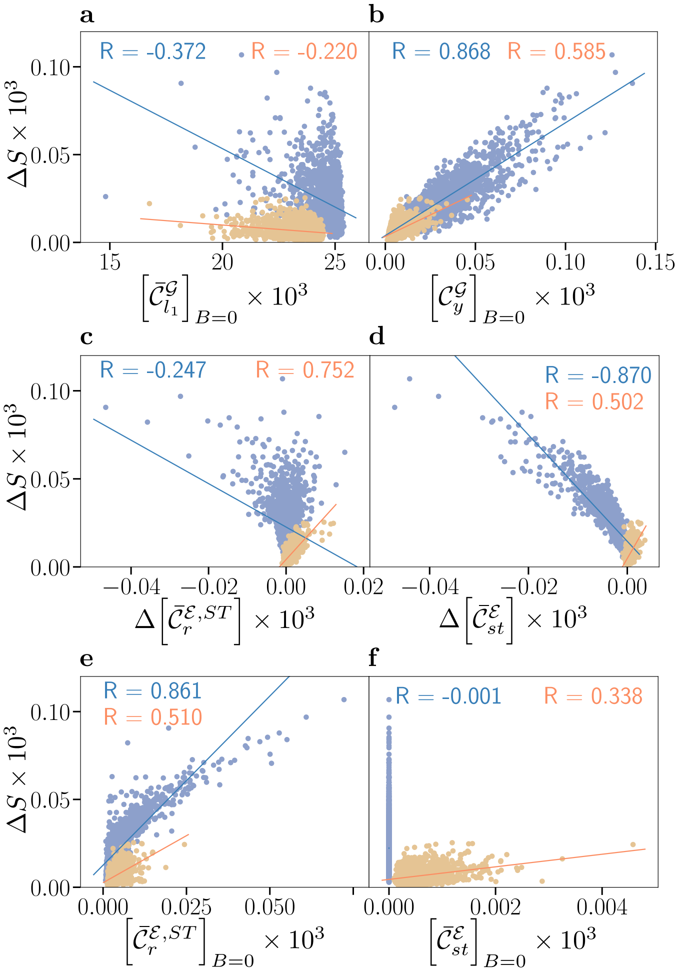

Fig. S7. Examples of the correlation between anisotropy Δ_S and global and electronic coherence measures is shown for systems G (blue) and C (peach). A linear fit has been plotted for each with associated correlation coefficient R.

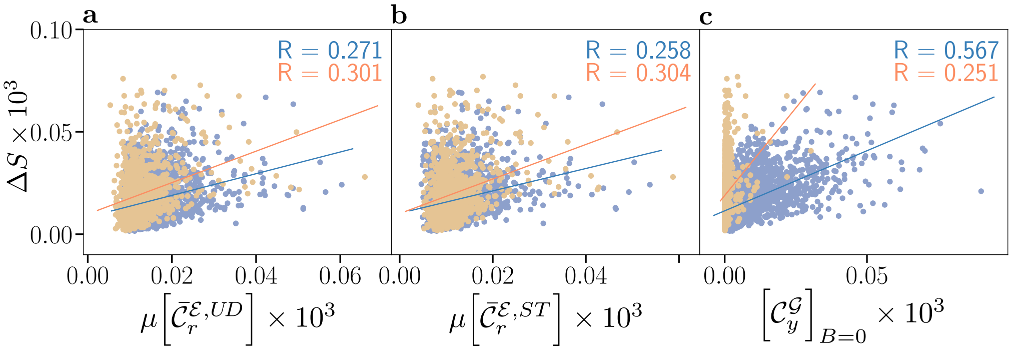

Fig. S8. Examples of the correlation between anisotropy Δ_S and global and electronic coherence measures is shown for systems A (blue) and B (peach). A linear fit has been plotted for each with associated correlation coefficient R.

Table S1. Hyperfine coupling parameters for nuclei in the flavin radical. The first two displayed parameters of N5 and N10, are also used for studies conducted using 5 nuclear spin systems.

Nucleus	Hyperfine interaction tensor (MHz)
N5	$\begin{bmatrix} -2.85 & 0.07 & -1.76 \\ 0.07 & -2.57 & 0.33 \\ -1.76 & 0.33 & 53.69 \end{bmatrix}$
N10	$\begin{bmatrix} -0.10 & 0.00 & -1.80 \\ 0.00 & -0.51 & -0.51 \\ 1.80 & -0.51 & 19.11 \end{bmatrix}$
H6	$\begin{bmatrix} -5.31 & -1.03 & 0.26 \\ -1.03 & -13.39 & -0.20 \\ 0.26 & -0.20 & -11.63 \end{bmatrix}$
Hβ1	$\begin{bmatrix} 8.49 & -0.88 & -1.22 \\ -0.88 & 5.57 & 0.30 \\ -1.22 & 0.30 & 5.35 \end{bmatrix}$
Hβ2	$\begin{bmatrix} 5.30 & 1.02 & -1.09 \\ 1.02 & 2.32 & -0.27 \\ -1.09 & -0.27 & 1.94 \end{bmatrix}$
H9	$\begin{bmatrix} 2.38 & 0.86 & -0.02 \\ 0.86 & 4.16 & 0.18 \\ -0.02 & 0.18 & 0.32 \end{bmatrix}$
3 $\times$ H7	$\begin{bmatrix} -3.78 & 0 & 0 \\ 0 & -3.78 & 0 \\ 0 & 0 & -3.78 \end{bmatrix}$
3 $\times$ H8	$\begin{bmatrix} 11.87 & 0 & 0 \\ 0 & 11.87 & 0 \\ 0 & 0 & 11.87 \end{bmatrix}$

Table S2. Hyperfine coupling parameters for nuclei in the tryptophan radical.

Nucleus	Hyperfine interaction tensor (MHz)
N1	$\begin{bmatrix} -1.94 & -0.05 & -0.21 \\ -0.05 & -2.30 & -0.44 \\ -0.21 & -0.44 & 19.16 \end{bmatrix}$
H1	$\begin{bmatrix} -2.14 & 6.32 & 0.17 \\ 6.32 & -18.90 & -0.04 \\ 0.17 & -0.04 & -14.75 \end{bmatrix}$
H2	$\begin{bmatrix} -21.18 & 4.42 & 0.16 \\ 4.42 & -4.33 & 0.11 \\ 0.16 & 0.11 & -15.99 \end{bmatrix}$
Hβ1	$\begin{bmatrix} 8.40 & -2.72 & -0.58 \\ -2.72 & 8.12 & 0.64 \\ -0.58 & 0.64 & 4.95 \end{bmatrix}$
Hβ2	$\begin{bmatrix} 27.49 & 0.81 & -1.79 \\ 0.81 & 24.00 & -0.35 \\ -1.79 & -0.35 & 22.85 \end{bmatrix}$
H4	$\begin{bmatrix} -6.44 & 0.79 & 0.27 \\ 0.79 & -23.04 & -0.04 \\ 0.27 & -0.04 & -17.06 \end{bmatrix}$
H5	$\begin{bmatrix} 2.51 & -1.02 & 0.02 \\ -1.02 & 4.52 & -0.08 \\ 0.02 & -0.08 & 0.65 \end{bmatrix}$
H6	$\begin{bmatrix} -14.6 & -4.82 & 0.08 \\ -4.82 & -5.12 & 0.02 \\ 0.08 & 0.02 & -11.26 \end{bmatrix}$
H7	$\begin{bmatrix} -1.05 & 1.17 & 0.09 \\ 1.17 & -8.50 & 0.07 \\ 0.09 & 0.07 & -7.36 \end{bmatrix}$

Table S3. Hyperfine coupling parameters for nuclei in the tyrosine radical.

Nucleus	Hyperfine interaction tensor (MHz)
H-*ortho* 1	$\begin{bmatrix} -25.96 & -7.79 & -0.04 \\ -7.79 & -11.84 & 0.02 \\ -0.04 & 0.02 & -21.77 \end{bmatrix}$
H-*ortho* 2	$\begin{bmatrix} -23.79 & 6.91 & -0.03 \\ 6.91 & -10.54 & -0.07 \\ -0.03 & -0.07 & -20.12 \end{bmatrix}$
H-*meta* 1	$\begin{bmatrix} 8.92 & -2.10 & -0.02 \\ -2.10 & 8.30 & 0.04 \\ -0.02 & 0.04 & 3.98 \end{bmatrix}$
H-*meta* 2	$\begin{bmatrix} 9.01 & 2.22 & -0.05 \\ 2.22 & 8.42 & 0.02 \\ -0.05 & 0.02 & 4.21 \end{bmatrix}$
Hβ1	$\begin{bmatrix} 6.07 & 2.09 & 0.44 \\ 2.09 & 2.79 & 0.01 \\ 0.44 & 0.01 & 1.85 \end{bmatrix}$
Hβ2	$\begin{bmatrix} 33.91 & -1.81 & 1.36 \\ -1.81 & 30.65 & -0.92 \\ 1.36 & -0.92 & 30.00 \end{bmatrix}$
Hγ	$\begin{bmatrix} -1.30 & -0.92 & -1.08 \\ -0.92 & -1.03 & 2.05 \\ -1.08 & 2.05 & -1.59 \end{bmatrix}$

Table S4. Parameter choices for systems A-H. Hyperfine interactions are chosen as either N5, N10 and a set of 3 random hyperfine interactions $\{A_{i,j}\}_{n=3}$, or 5 random hyperfine interactions assigned to radical A$^\bullet$ and B$^\bullet$ as $\{A_{i,j}\}_{n=2}$ and $\{A_{i,j}\}_{n=3}$, or $\{A_{i,j}\}_{n=5}$. Under the hyperfine interactions column, radical A$^\bullet$ is referred to as 1, and radical B$^\bullet$ is referred to as 2 for clarity.

System	Hyperfine interactions	Lifetime k^{-1} (μs)
A	1: N5, N10<br>2: $\{A_{i,j}\}_{n=3}$	1
B	1: N5, N10<br>2: $\{A_{i,j}\}_{n=3}$	10
C	1: $\{A_{i,j}\}_{n=2}$<br>2: $\{A_{i,j}\}_{n=3}$	1
D	1: $\{A_{i,j}\}_{n=2}$<br>2: $\{A_{i,j}\}_{n=3}$	10
E	1: N5, N10, $\{A_{i,j}\}_{n=3}$<br>2: none	1
F	1: N5, N10, $\{A_{i,j}\}_{n=3}$<br>2: none	10
G	1: $\{A_{i,j}\}_{n=5}$<br>2: none	1
H	1: $\{A_{i,j}\}_{n=5}$<br>2: none	10

Code Snippets

Spin correlation tensor computation. The following CUDA kernel, and subsequent Python routines, were used in the computation of spin correlation tensors (SCTs). corr_fun_cuda takes as inputs the matrix representations of $\hat{S}_{i,x}$, $\hat{S}_{i,y}$ and $\hat{S}_{i,z}$ in the eigenbasis of the Hamiltonian and the associated eigen-frequencies, together with the time step and number of steps.

```
import numpy as np

import pycuda.driver as drv
from pycuda.compiler import SourceModule

import sct_kernels


def mk_kernels():

    mod = SourceModule("""
        typedef double ftype;
        typedef double2 f2type;
        __global__ void column_sum_re(const f2type* __restrict__ input,
                                      ftype* __restrict__ per_block_results,
                                      const size_t lda, const size_t n)
        {
            extern __shared__ ftype sdata[];

            ftype x = 0.0;
            const f2type * p = &input[blockIdx.x * lda];
            for(int i=threadIdx.x; i < n; i += blockDim.x) {
                x += p[i].x;
            }
            sdata[threadIdx.x] = x;
            __syncthreads();

            for(int offset = blockDim.x / 2; offset > 0; offset >>= 1) {
                if(threadIdx.x < offset) {
                    sdata[threadIdx.x] += sdata[threadIdx.x + offset];
                }
                __syncthreads();
            }

            // thread 0 writes the final result
            if(threadIdx.x == 0) {
                per_block_results[blockIdx.x] = sdata[0];
            }

        }

        __global__ void cplx_multiply_update(const f2type* __restrict__ multiplier,
                                      f2type* __restrict__ values,
                                      const size_t lda, const size_t n)
        {

            f2type * p = &values[blockIdx.y * lda];
            ftype a, b, c, d;
            for (int i = blockIdx.x * blockDim.x + threadIdx.x;
                 i < n;
                 i += blockDim.x * gridDim.x) {
                a = p[i].x;
                b = p[i].y;
                c = multiplier[i].x;
                d = multiplier[i].y;
                p[i].x = a*c - b*d;
                p[i].y = a*d + b*c;
```

```python
            }
        }
    """)

    reduction_kernel = mod.get_function("column_sum_re")
    reduction_kernel.prepare("PPNN")

    propagation_kernel = mod.get_function("cplx_multiply_update")
    propagation_kernel.prepare("PPNN")

        return reduction_kernel, propagation_kernel

def _init_propagators(X, Y, Z, energies, dt):
    n = X.shape[0]
    ind = np.triu_indices(n, k=1) # off-diagonal, upper triangular elements only
    ab = np.empty(((n * n - n) // 2, 9), dtype=np.complex128, order='F')
    xyz = [np.asarray(op[ind]).reshape(-1) for op in (X, Y, Z)]
    for i in range(3):
        for j in range(3):
            ab[:,i*3+j] = xyz[i].conj() * xyz[j] # A[ind].conj() = (A.T)[ind]
    p = np.exp(-1j * dt * energies)
    p = p[ind[0]].conj() * p[ind[1]]
    return ab, p


def corr_fun_cuda(X, Y, Z, energies, dt, m, kernels=None):

    if kernels is None:
        reduction_kernel, propagation_kernel = mk_kernels()
    else:
        reduction_kernel, propagation_kernel = kernels

    ab, p = _init_propagators(X, Y, Z, energies, dt)
    corr_fun = np.zeros((m, 9), dtype=np.float64)
    corr_fun[0,:] = ab.sum(axis=0).real # one halve only
    n = ab.shape[0]

    grid_red = (9, 1, 1)
    block_red = (1024, 1, 1)
    shared_red = block_red[0]*8

    block_prop = (1024, 1, 1)
    grid_prop = ((n+block_prop[0]-1)//block_prop[0], 9, 1)

    d_ab = drv.mem_alloc(ab.nbytes)
    drv.memcpy_htod(d_ab, np.asfortranarray(ab))
    d_p = drv.mem_alloc(p.nbytes)
    drv.memcpy_htod(d_p, p)
    c = np.empty((9,), dtype=np.float64)
    d_c = drv.mem_alloc(c.nbytes)

    for i in range(1, m):
        propagation_kernel.prepared_call(grid_prop, block_prop,
            d_p, d_ab, n, n)
        reduction_kernel.prepared_call(grid_red, block_red,
            d_ab, d_c, n, n, shared_size=shared_red)
        drv.memcpy_dtoh(c, d_c)
        corr_fun[i] = c

    corr_fun *= 2.
```

```
    xyz = (X, Y, Z)
    for i in range(3):
        for j in range(3):
            corr_fun[:,i*3+j] += (np.diag(xyz[i]).dot(np.diag(xyz[j]))).real

    return corr_fun
```

Recombination yield computation. The following code was used to compute the recombination yield directly and subsequently implemented to obtain the global measure of coherence introduced in the main text. singletYield_offdiag calculates the singlet recombination yield due to the off-diagonal elements of $\hat{P}_S$ in the eigenbasis of the Hamiltonian. It takes as its inputs the recombination rate constant and the matrix representations of $\hat{S}_{i,x}$, $\hat{S}_{i,y}$ and $\hat{S}_{i,z}$ in the eigenbasis of the Hamiltonian and the associated eigen-frequencies for both radicals.

```
import numpy as np
import pycuda.driver as cuda
import pycuda.autoinit
from pycuda.compiler import SourceModule
import numba

def compile_kernel():
    mod = SourceModule("""

        #include "cuda_runtime.h"
        #include "device_launch_parameters.h"

        // typedef float ftype;
        // typedef float2 ctype;
        typedef double ftype;
        typedef double2 ctype;

        /* ------------------------------------------------------------------------ */

        /* Complex arithmetics (not guarded against overflow) */


        __host__ __device__ static __inline__ ctype mkCplx(ftype re, ftype im) {
            ctype cplx = {.x = re, .y = im};
            return cplx;
        }

        __host__ __device__ static __inline__ ctype cplxTimes(ctype a, ctype b) {
            return mkCplx(a.x * b.x - a.y * b.y, a.x * b.y + a.y * b.x); // unguarded against overflow!
        }

        __host__ __device__ static __inline__ ctype cplxAdd(ctype a, ctype b) {
            return mkCplx(a.x + b.x, a.y + b.y);
        }

        __host__ __device__ static __inline__ ftype cplxAbs2(ctype a) {
            return a.x*a.x + a.y*a.y; // unguarded against overflow!
        }

        __host__ __device__ static __inline__ ftype kernel(ftype k2, ctype xa, ctype ya, ctype za, ftype da, ctype xb,
            ftype delta = da + db;
            return cplxAbs2(cplxAdd(cplxTimes(xa,xb),
                                    cplxAdd(cplxTimes(ya,yb),
                                            cplxTimes(za,zb)))) * (k2 / (delta*delta + k2));
        }


        /* ------------------------------------------------------------------------ */
```

```
    // static const int warpSize = 32; // in device_launch_parameters.h

    __device__ ftype sum_shared_single_warp(volatile ftype* sdata) {
        int idx = threadIdx.x % warpSize; //the lane index in the warp
        if(idx < 16) {
        sdata[idx] += sdata[idx+16];
        sdata[idx] += sdata[idx+8];
        sdata[idx] += sdata[idx+4];
        sdata[idx] += sdata[idx+2];
        sdata[idx] += sdata[idx+1];
        }

    __global__ void calcYield_kernel_v2(
        const ftype k2,
        const int na,
        const ctype* __restrict__ sax, const ctype* __restrict__ say, const ctype* __restrict__ saz,
        const ftype* __restrict__ deltaa,
        const int nb,
        const ctype* __restrict__ sbx, const ctype* __restrict__ sby, const ctype* __restrict__ sbz,
        const ftype* __restrict__ deltab,
        ftype* out) {

        extern __shared__ ftype sdata[]; //  __shared__ ftype sdata[blockSize];

        const int tidx = threadIdx.x;
        const int blockSize = blockDim.x; // max blocksize = 32*32 = 1024
        const int gridSize = blockSize*gridDim.x;
        ctype xa, ya, za;
        ftype la;
        double y = 0;

        for(int i = tidx + blockIdx.x*blockSize; i < na; i += gridSize) {
            xa = sax[i];
            ya = say[i];
            za = saz[i];
            la = deltaa[i];
            for(int j = 0; j < nb; ++j)
                y += kernel(k2, xa, ya, za, la,
                                sbx[j], sby[j], sbz[j], deltab[j]);
        }
        sdata[tidx] = y;

        sum_shared_single_warp(&sdata[tidx & ~(warpSize-1)]);
        __syncthreads();
        if(tidx < warpSize) { //first warp only
            sdata[tidx] = tidx*warpSize<blockSize ? sdata[tidx*warpSize] : 0;
            sum_shared_single_warp(sdata);
            if(tidx == 0)
                out[blockIdx.x] = sdata[0];
        }

        return;
    }
    """)

kernel = mod.get_function("calcYield_kernel_v2")
ctype = np.complex128
# ctype = np.complex64
ftype = np.empty(0, dtype=ctype).real.dtype
```

```python
    return kernel, ctype, ftype


_kernel, _ctype, _ftype = compile_kernel()

def singletYield_offdiag_kernel(kr, z, Sxyz1el, delta1, Sxyz2el, delta2):

    ctype = _ctype
    ftype = _ftype

    blockSize = 1024;
    nrBlocks = (len(delta1) + (blockSize - 1)) // blockSize
    sharedmem = ftype.itemsize * blockSize
    print(nrBlocks, blockSize, len(delta1), len(delta2))

    def toGPU(a):
        a_gpu = cuda.mem_alloc(a.size * a.dtype.itemsize)
        cuda.memcpy_htod(a_gpu, a)
        return a_gpu

    d_Sxyz1el = [toGPU(x.astype(ctype)) for x in Sxyz1el]
    d_Sxyz2el = [toGPU(x.astype(ctype)) for x in Sxyz2el]
    d_delta1 = toGPU(delta1.astype(ftype))
    d_delta2 = toGPU(delta2.astype(ftype))

    d_out = cuda.mem_alloc(nrBlocks * ftype.itemsize)

    kr2 = kr*kr

    _kernel(ftype.type(kr2),
            np.intc(len(delta1)), *d_Sxyz1el, d_delta1,
            np.intc(len(delta2)), *d_Sxyz2el, d_delta2,
            d_out,
            block=(blockSize,1,1), grid=(nrBlocks,1,1), shared=sharedmem)

    out = np.empty(nrBlocks, dtype=ftype)
    cuda.memcpy_dtoh(out, d_out)
    y = np.sum(out)/z*2

    for op in d_Sxyz1el: op.free()
    for op in d_Sxyz2el: op.free()
    d_delta1.free()
    d_delta2.free()
    d_out.free()

    return y



def triu_elements(Sxyz, lambdas, k):
    d = Sxyz.shape[-1]
    i, j = np.triu_indices(d, k)
    return [op[i,j] for op in Sxyz], lambdas[j] - lambdas[i]
def diag_elements(Sxyz, lambdas):
    d = Sxyz.shape[-1]
    i, j = np.diag_indices(d)
    return [op[i,j] for op in Sxyz], np.zeros(d, dtype=lambdas.dtype)
def all_elements(Sxyz, lambdas):
    d = Sxyz.shape[-1]
    i, j = np.meshgrid(np.arange(d), np.arange(d))
    i = i.reshape(-1)
```

```python
    j = j.reshape(-1)
    return [op[i,j] for op in Sxyz], lambdas[j] - lambdas[i]

def singletYield_offdiag(kr, Sxyz1, lambda1, Sxyz2, lambda2, skipDiagA=False):

    # off diagonal elements of A x B  ==  A_tri1 x B_all  +  A_diag x B_tri1

    Sxyz1el, delta1 = triu_elements(Sxyz1, lambda1, 1) # strictly upper triangular part
    Sxyz2el, delta2 = all_elements(Sxyz2, lambda2)

    z = len(lambda1)*len(lambda2) // 4

    y = singletYield_offdiag_kernel(kr, z, Sxyz1el, delta1, Sxyz2el, delta2)

    if not skipDiagA:
        # odd number of H in A
        Sxyz1el, delta1 = diag_elements(Sxyz1, lambda1)
        Sxyz2el, delta2 = triu_elements(Sxyz2, lambda2, 1) # strictly upper triangular part
        y += singletYield_offdiag_kernel(kr, z, Sxyz1el, delta1, Sxyz2el, delta2)

    return y
```

References

1. Atkins, C., Bajpai, K., Rumball, J., Kattnig, D. R., On the optimal relative orientation of radicals in the cryptochrome magnetic compass. *The J. Chem. Phys.* **151**, 065103 (2019).
2. Manolopoulos, D. E., Hore, P. J., An improved semiclassical theory of radical pair recombination reactions. *The J. Chem. Phys.* **139**, 124106 (2013).
3. Schulten, K., Wolynes, P. G., Semiclassical description of electron spin motion in radicals including the effect of electron hopping. *The J. Chem. Phys.* **68**, 3292–3297 (1978).
4. Cai, J., Plenio, M. B., Chemical compass model for avian magnetoreception as a quantum coherent device. *Phys. Rev. Lett.* **111**, 230503 (2013).
5. Timmel, C. R., Till, U., Brocklehurst, B., Mclauchlan, K. A., Hore, P. J., Effects of weak magnetic fields on free radical recombination reactions. *Mol. Phys.* **95**, 71–89 (1998).
6. Wong, S. Y., Solov'yov, I. A., Hore, P. J., Kattnig, D. R., Nuclear polarization effects in cryptochrome-based magnetoreception. *The J. Chem. Phys.* **154**, 035102 (2021).
